# Robust chitinolytic activity of crab-eating monkey (*Macaca fascicularis*) acidic chitinase under a broad pH and temperature range

**DOI:** 10.1038/s41598-021-95010-w

**Published:** 2021-07-29

**Authors:** Maiko Uehara, Eri Tabata, Mikoto Okuda, Yukari Maruyama, Vaclav Matoska, Peter O. Bauer, Fumitaka Oyama

**Affiliations:** 1 Department of Chemistry and Life Science, Kogakuin University, Hachioji, Tokyo 192-0015 Japan; 2 Research Fellow of Japan Society for the Promotion of Science (PD), Chiyoda-ku, Koujimachi, Tokyo, 102-0083 Japan; 3 Laboratory of Molecular Diagnostics, Department of Clinical Biochemistry, Hematology and Immunology, Homolka Hospital, Roentgenova 37/2, Prague, 150 00 Czech Republic; 4 Bioinova Ltd., Videnska 1083, Prague, 142 20 Czech Republic

**Keywords:** Enzymes, Hydrolases, Carbohydrates, Polysaccharides, Biochemistry, Zoology, Animal physiology

## Abstract

Diet of the crab-eating monkey (*Macaca fascicularis*) consists of both plants and animals, including chitin-containing organisms such as crabs and insects. This omnivorous monkey has a high expression of acidic chitinase (CHIA) in the stomach and here**,** we report on its enzymatic properties under different conditions. When we compared with *Mus musculus* CHIA (*Mm*-CHIA), *Macaca fascicularis* CHIA (*Mf*-CHIA) exhibits higher chitinolytic activity at broad pH (1.0–7.0) and temperature (30–70 ℃) range. Interestingly, at its optimum pH (5.0), *Mf*-CHIA showed the highest activity at 65 °C while maintaining it at robust levels between 50 and 70 °C. The degradation efficiency of *Mf*-CHIA was superior to *Mm*-CHIA toward both polymeric chitin as well as an artificial chromogenic substrate. Our results show that unique features of *Mf*-CHIA including its thermostability warrant the nomination of this enzyme for potential agricultural and biomedical applications.

## Introduction

Chitin, a polymer of *N*-acetyl-D-glucosamine (GlcNAc), is the second most abundant polysaccharide in nature^1,2^. It is the main component of crustaceans and insects’ exoskeletons, the microfilarial sheaths of parasitic nematodes and fungal cell walls^1–3^.

Although mammals do not produce chitin, they express chitinases in their tissues. Many species have genes that encode two active chitinases, chitotriosidase (CHIT1) and acidic chitinase (we referred to as “CHIA”; also called acidic mammalian chitinase, AMCase)^2–4^. CHIT1 was the first mammalian chitinase to be purified and cloned^5–7^ and CHIA was identified as a compensatory enzyme for CHIT1^8,9^. Recently, the application of chitinases and acetyl-glucosaminidases in industrial use was discussed^10–12^.

CHIA plays an essential role in the pathophysiological condition. CHIA expression levels are markedly altered in various diseases such as asthma, allergic inflammation, dry eye syndrome, and gastric cancer^13–19^. Moreover, several genetic variants of CHIA are associated with bronchial asthma in humans^20–23^. Recent studies using CHIA-deficient mice have shown that CHIA protects the lung from the damaging effects of widespread polysaccharides, including chitin^24^. Furthermore, CHIA functions as a critical initiator of the protective immune response to gastrointestinal nematodes in the host gastrointestinal tract^25^.

The crab-eating monkey (*Macaca fascicularis*; Old World monkey) provides a crucial nonhuman primate animal model for biomedical research^26,27^. This primate's name came from its feeding habits when different chitin-containing organisms, including crabs, comprise the central part of its diet^28^. We have previously performed gene expression analysis and found that the monkey expresses a high level of CHIA mRNA in the stomach^29^. We have shown that mouse, chicken and pig Chia are major protease-resistant glycosidases in the respective digestive systems^30,31^. Furthermore, common marmoset (New World monkey) CHIA is most active at pH 2.0 and degrades chitin and mealworm shells into GlcNAc dimers [(GlcNAc)_2_] under gastrointestinal conditions^32^.

In this study, we aimed to investigate *Macaca fascicularis* CHIA (*Mf*-CHIA) and found that it maintained high chitinolytic activity under a broad range of pH and thermal conditions. We also discuss whether or not this enzyme is suitable for agricultural, biomedical and industrial purposes.

## Results

### Preparation of recombinant CHIA

**Figure 1:**
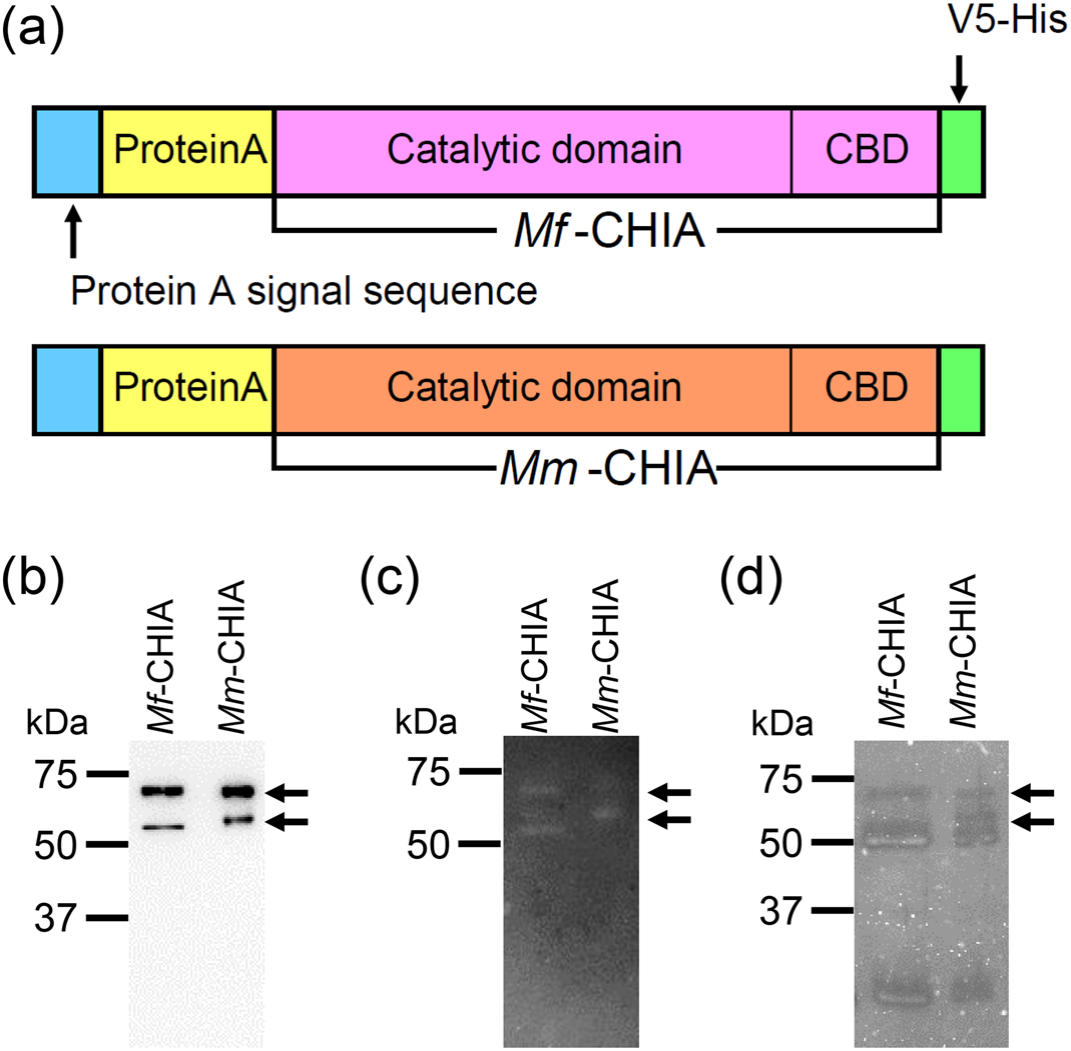
Schematic representations of the *E. coli*-expressed *Mf*-CHIA and *Mm*-CHIA fusion proteins (Protein A-*Mf*-CHIA or *Mm*-CHIA-V5-His). (**a**) *E. coli*-expressed Protein A-*Mf*-CHIA or *Mm*-CHIA-V5-His. The estimated size for Protein A-*Mf*-CHIA or *Mm*-CHIA-V5-His is 68 kDa. Analysis of the recombinant proteins by Western blot using the anti-V5 antibody (**b**). (**c**) Zymogram of *Mf*-CHIA and *Mm*-CHIA. (**d**) SYPRO Ruby stain. The images of (**b**), (**c**) and (**d**) were cropped from red dotted lines on original full-length gel images shown in Supplementary Fig. S2, S3, S4 and S5.

The preparation of *Mus musculus* CHIA (*Mm*-CHIA) as a fusion protein (Protein A-*Mm*-CHIA-V5-His) using the pEZZ18 system in *E. coli* has been described previously^33^. Here, we expressed *Mf*-CHIA using the same protocol (Fig. 1a and Supplementary Fig. S1). The estimated size for Protein A-*Mf*-CHIA or *Mm*-CHIA-V5-His is 68 kDa.

The recombinant *Mf*-CHIA and *Mm*-CHIA enzymes were analyzed by SDS–polyacrylamide gel electrophoresis (PAGE), followed by Western blot using an anti-V5 antibody. We detected the proteins as major and minor bands of around 68 and 55 kDa, respectively (Fig. 1b and Supplementary Fig. S2). The signal intensities of both bands were equal between *Mf*-CHIA and *Mm*-CHIA (Supplementary Fig. S3) and zymographic analysis revealed chitinolytic activity in all of them (Fig. 1c and Supplementary Fig.S4). This suggests that the obtained protein samples contain full length (Protein A-CHIA-V5-His, 68 kDa) and truncated form (CHIA-V5-His, 55 kDa) of the recombinant molecules.

The gel with separated samples was also stained by SYPRO Ruby (Fig. 1d and Supplementary Fig. S5), detecting additional bands not visible by Western blot. We considered the bands around 55 kDa with chitinolytic activity as truncated CHIA forms. They may have contained contaminating proteins unrelated to CHIA with no chitinolytic activity. After obtaining Protein A-*Mf*-CHIA-V5-His sample by IgG Sepharose separation, we attempted to further purify the recombinant protein using anion change chromatography. However, this step markedly reduced the protein yield while not providing additional improvement. Thus, *Mf*-CHIA and *Mm*-CHIA were prepared simply by IgG Sepharose columns and used as mixture proteins in this study.

### pH dependence of *Mf*-CHIA activity

To gain insight into the functioning of *E. coli*-expressed *Mf*-CHIA, we first examined its chitinolytic activity using 4-nitrophenyl *N,N′*-diacetyl-β-D-chitobioside [4-NP-(GlcNAc)_2_] chromogenic substrate at different pH in 0.1 M Gly-HCl (pH 1.0–3.0) or McIlvaine’s (pH 2.0–8.0) buffers for 60 min at 37 °C. We set the concentration of the enzymes to provide a 20% rate of substrate consumption as compared to the initial solution (Supplementary Fig. S6). The resulting *Mf*-CHIA and *Mm*-CHIA concentrations in the reactions were 32.82 ng/μL and 35.57 ng/μL, respectively. The chitinolytic activities were expressed with subtraction of the background obtained in blank experiments with no enzymes.

It has been reported that the apparent chitinolytic activity of human CHIA may decrease by its transglycosylation activity when the substrate [4-methylumbelliferyl chitobioside, 4-MU-(GlcNAc)_2_] has higher concentrations^34^. Here, we used 4-NP-(GlcNAc)_2_ and compared its degradation at concentrations ranging from 20 μM to 400 μM. The substrate degradation intensified with its increasing initial concentration and no degradation reduction was observed even at the highest initial concentration (400 μM) (Supplementary Fig. S6). Thus, we confirmed that our chitinase enzymatic assay was not affected by transglycosylation.

**Figure 2:**
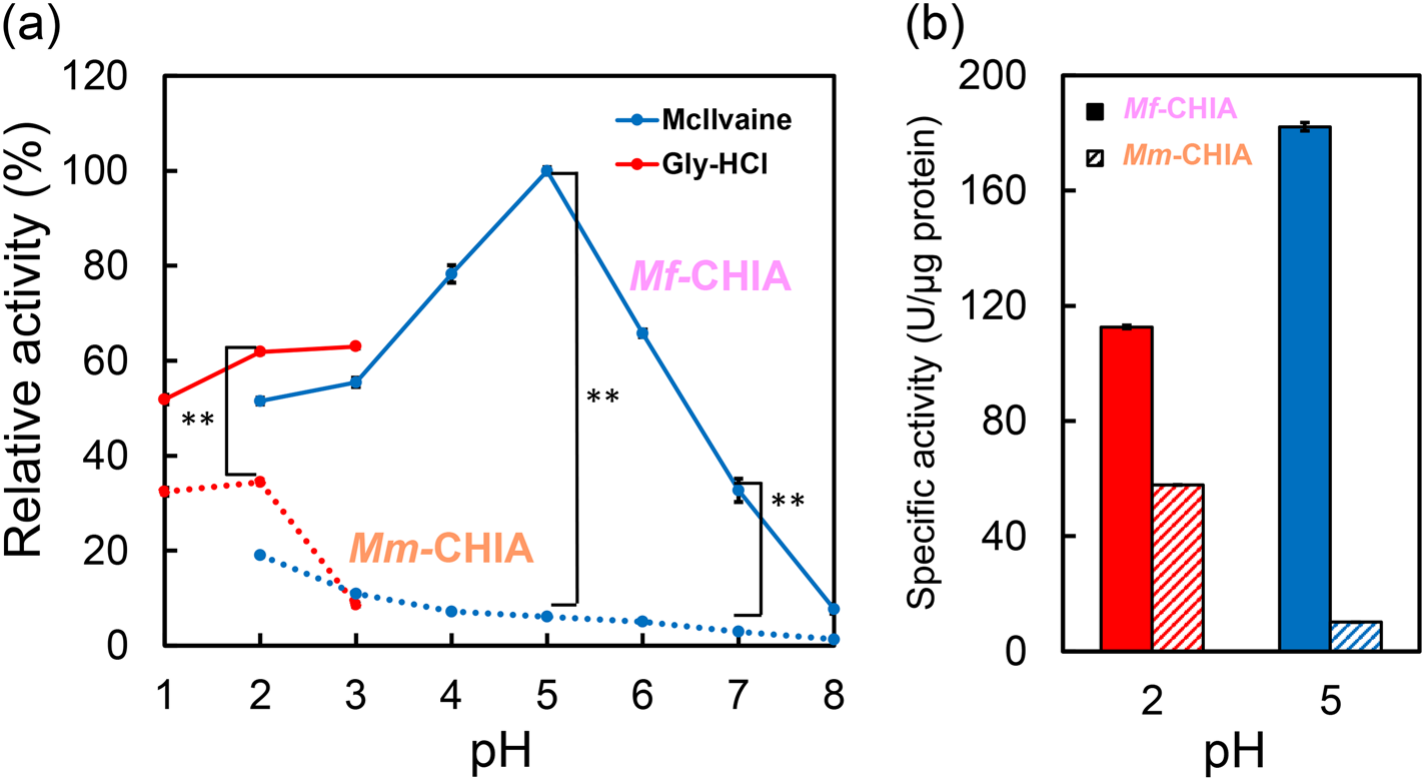
Optimal pH for *Mf*-CHIA and comparison with *Mm*-CHIA. The chitinolytic activity of *Mf*-CHIA and *Mm*-CHIA was measured using 4-NP-(GlcNAc)_2_. (**a**) Solid lines, *Mf*-CHIA; dotted lines, *Mm*-CHIA. The chitinase activity was analyzed in Gly-HCl buffer (pH 1.0 to 3.0) or McIlvaine's buffer (pH 2.0 to 8.0) at 37 °C for 60 min. Red lines, Gly-HCl buffer; blue lines, McIlvaine’s buffer. We show relative activity when the *Mf*-CHIA activity level at pH 5.0 was set to 100%. (**b**) Showing specific enzymatic activity, we compared *Mf*-CHIA with *Mm*-CHIA at their respective optimal pH (*Mf*-CHIA at pH 5.0, *Mm*-CHIA at pH 2.0). Filled bars, *Mf*-CHIA; hatched bars, *Mm*-CHIA. The chitinolytic activities are expressed with the subtraction of the blank experiments. Error bars represent mean ± standard deviation from a single experiment conducted in triplicate. ** *p* < 0.01.

The peak activity was observed at pH 5.0 with high levels between pH 1.0–6.0 and being present even at pH 7.0 (Fig. 2a). When the *Mf*-CHIA activity level at pH 5.0 was set to 100%, the relative activity at pH 2.0 (Gly-HCl buffer), pH 2.0 (McIlvaine’s buffer) and pH 7.0 (McIlvaine’s buffer) were 62%, 52% and 31%, respectively. Notably, the recombinant *Mf*-CHIA had properties very similar to the native enzyme from stomach extracts of the crab-eating monkey for the pH preference^29^.

When compared to *Mm*-CHIA, *Mf*-CHIA had higher activity at each condition. There was a threefold difference in their peak activities *Mf*-CHIA at pH 5.0, *Mm*-CHIA at pH 2.0 being 182.1 U/μg and 57.83 U/μg protein, respectively (Fig. 2b). In addition, *Mf*-CHIA was also 2×, 16×, and 10× more active at pH 2.0, 5.0, and 7.0, respectively, than the mouse enzyme. Thus, *Mf*-CHIA's chitinolytic activity properties differ from other animals reported previously^30–33,35,36^ and are rather similar to human CHIA, at least in regard to pH^23,34,37^.

### Temperature dependence of *Mf*-CHIA activity

**Figure 3:**
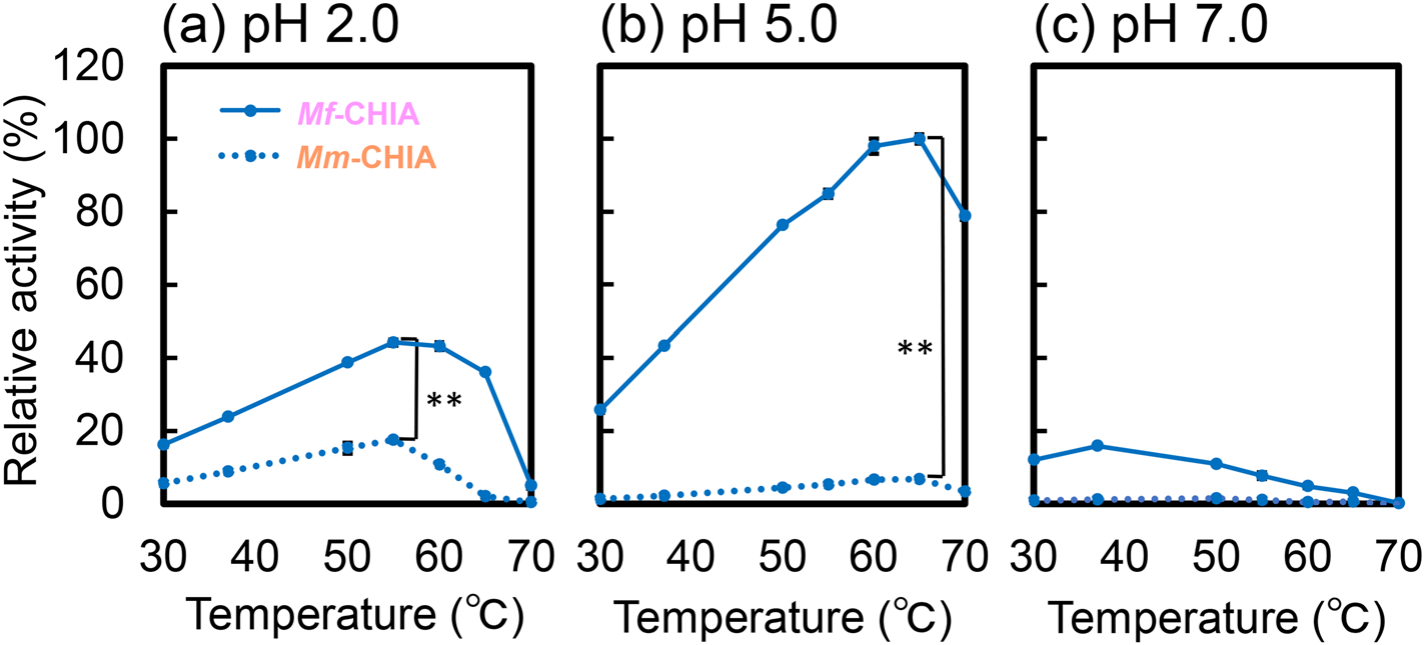
The optimal temperature of *Mf*-CHIA and comparison with *Mm*-CHIA. The chitinase activity was investigated between 30 and 80 °C for 60 min in McIlvaine’s buffer. (**a**) pH 2.0, (**b**) pH 5.0 and (**c**) pH 7.0. Solid lines, *Mf*-CHIA; dotted lines, *Mm*-CHIA. We show relative activities of both enzymes where the peak activity of *Mf*-CHIA under optimal conditions (pH 5.0 and 65 °C) was set to 100%. The chitinolytic activities are expressed with the subtraction of the blank experiments. Error bars represent mean ± standard deviation from a single experiment conducted in triplicate. **p < 0.01.

The effect of temperature on enzyme activity was determined in McIlvaine’s buffer at pH 2.0, 5.0 or 7.0 and 30–70 °C for 60 min. Figure 3 shows relative activities of both enzymes where the peak activity of *Mf*-CHIA under optimal conditions (pH 5.0 and 65 °C) was set to 100%. At pH 2.0, the optimal value for *Mm*-CHIA, *Mf*-CHIA was most active at 55 ℃ (Fig. 3a). As shown in Fig. 3b (pH 5.0), the reaction rate increased with the temperature and reached the maximum level at 65 °C. At pH 7.0, *Mf*-CHIA reached the peak activity at 37 ℃ and then rapidly decreased with increasing temperature (Fig. 3c). More detailed comparison of the temperature-dependence between *Mf*-CHIA and *Mm*-CHIA is shown in Supplementary Fig. S7. At pH 2.0, *Mf*-CHIA retained high activity at 65 ℃, whereas in *Mm*-CHIA, the activity markedly dropped at > 60 ℃. At pH 5.0, the monkey enzyme maintained its high activity even at 70 ℃ (approximately 80%). At pH 7.0, the activity of *Mf*-CHIA dropped at > 50 ℃, on the other hand, *Mm*-CHIA retained its (relatively low) activity at 50 ℃.

Overall, chitinolytic activity of *Mf*-CHIA significantly exceeded that of *Mm*-CHIA at each tested condition (Fig. 3). while demonstrating high thermostability.

### pH stability of the *Mf*-CHIA

**Figure 4:**
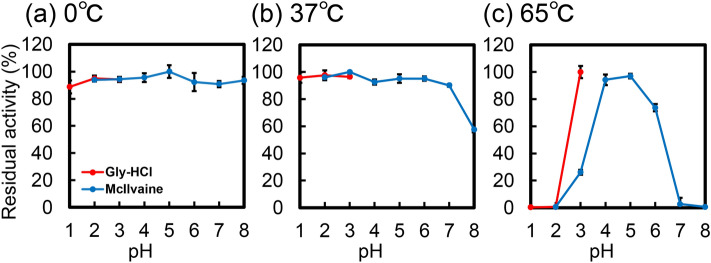
pH stability of *Mf*-CHIA. *Mf*-CHIA was incubated for 1 h at 0 °C, 37 °C or 65 °C in Gly-HCl buffer (pH 1.0 to 3.0), McIlvaine's buffer (pH 2.0 to 8.0). After the pre-incubation at the indicated pH, the residual activity was measured at pH 5.0 (optimal pH condition of monkey CHIA) in McIlvaine’s buffer. Red lines, Gly-HCl buffer; blue lines, McIlvaine’s buffer. (**a**) 0 °C, (**b**) 37 °C and (**c**) 65 °C. We show the relative activity when the highest residual activity of *Mf*-CHIA was set to 100%. The chitinolytic activities are expressed with the subtraction of the blank experiments. Error bars represent mean ± standard deviation from a single experiment conducted in triplicate.

Next, we aimed to determine the pH stability of the enzyme at different temperatures. *Mf*-CHIA was pre-incubated at 0, 37 or 65 ℃ for 60 min at pH 1.0–8.0. Pre-incubation was followed by enzymatic activity analysis at 37 °C and pH 5.0 for 60 min. We show the relative activity when the highest residual activity of *Mf*-CHIA was set to 100%. As shown in Fig. 4a, *Mf*-CHIA remained stable over a broad pH range (pH 1.0–8.0), during the 1-h pre-incubation at 0 ℃. This treatment caused no measurable decrease in chitinase activity.

Pre-incubation at 37 ℃ resulted in maintained activity at pH 1.0 to 7.0 (Fig. 4b) and that at 65 ℃ resulted in inactivation at pH 1.0–2.0 and 7.0–8.0 (Fig. 4c). Thus, the *E. coli-*expressed *Mf*-CHIA is stable under acidic conditions.

### Thermal stability of the *Mf*-CHIA

**Figure 5:**
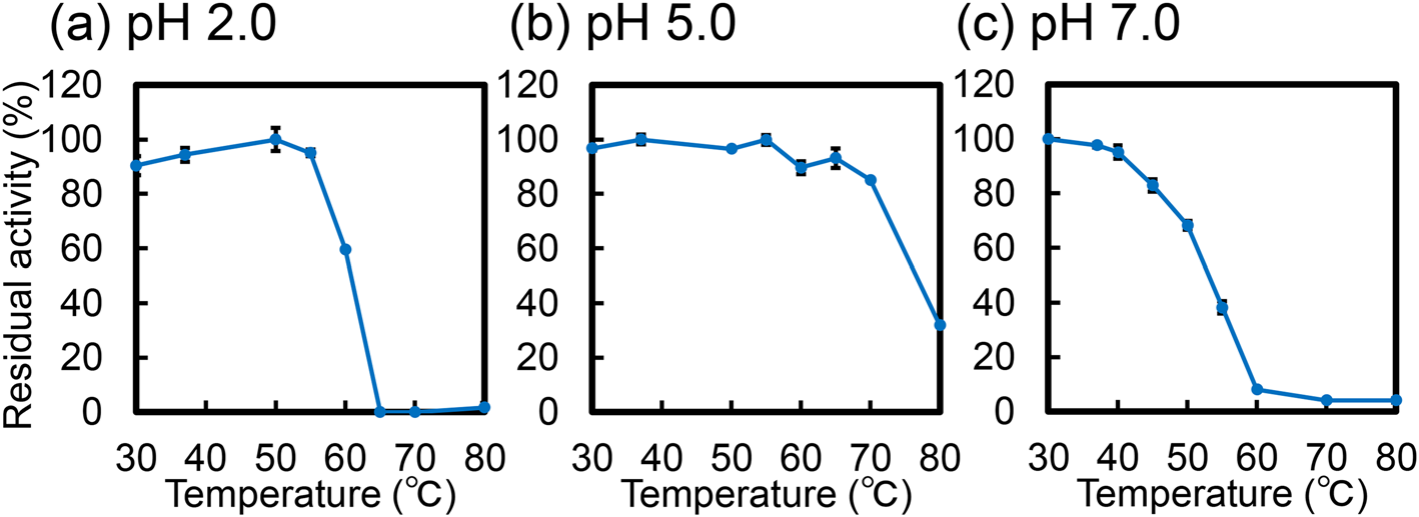
Temperature stability of *Mf*-CHIA. *Mf*-CHIA were incubated at pH 2.0, 5.0 and 7.0 for 30 min in McIlvaine’s buffer for 15 min between 30 and 80 °C. After cooling on ice, the residual activity was measured at pH 5.0 (optimal pH condition of monkey CHIA) in McIlvaine’s buffer. (**a**) pH 2.0, (**b**) pH 5.0 and (**c**) pH 7.0. We show the relative activity when the highest residual activity of *Mf*-CHIA was set to 100%. The chitinolytic activities are expressed with the subtraction of the blank experiments. Error bars represent mean ± standard deviation from a single experiment conducted in triplicate.

We assessed the *Mf*-CHIA's thermal stability with pre-incubation of the samples at pH 2.0, 5.0 or 7.0 for 30 min at 30–80 °C. The residual activity was measured using the chromogenic substrate at 37 °C and pH 5.0 for 60 min. We show the relative activity when the highest residual activity of *Mf*-CHIA was set to 100%. Pre-incubation at pH 2.0 resulted in the enzyme's deactivation above 60 ℃, where it retained 60% of its peak activity (Fig. 5a). The enzyme also remained stable to up to 70 ℃ at pH 5.0 and at 80 ℃, there was still 31% activity present (Fig. 5b). At pH 7.0, *Mf*-CHIA was stable to up to 40 ℃ (Fig. 5c). Thus, *Mf*-CHIA is stable at 30–70 ℃ depending on the pH conditions.

### Degradation of polymeric chitin by *Mf*-CHIA and *Mm*-CHIA

**Figure 6:**
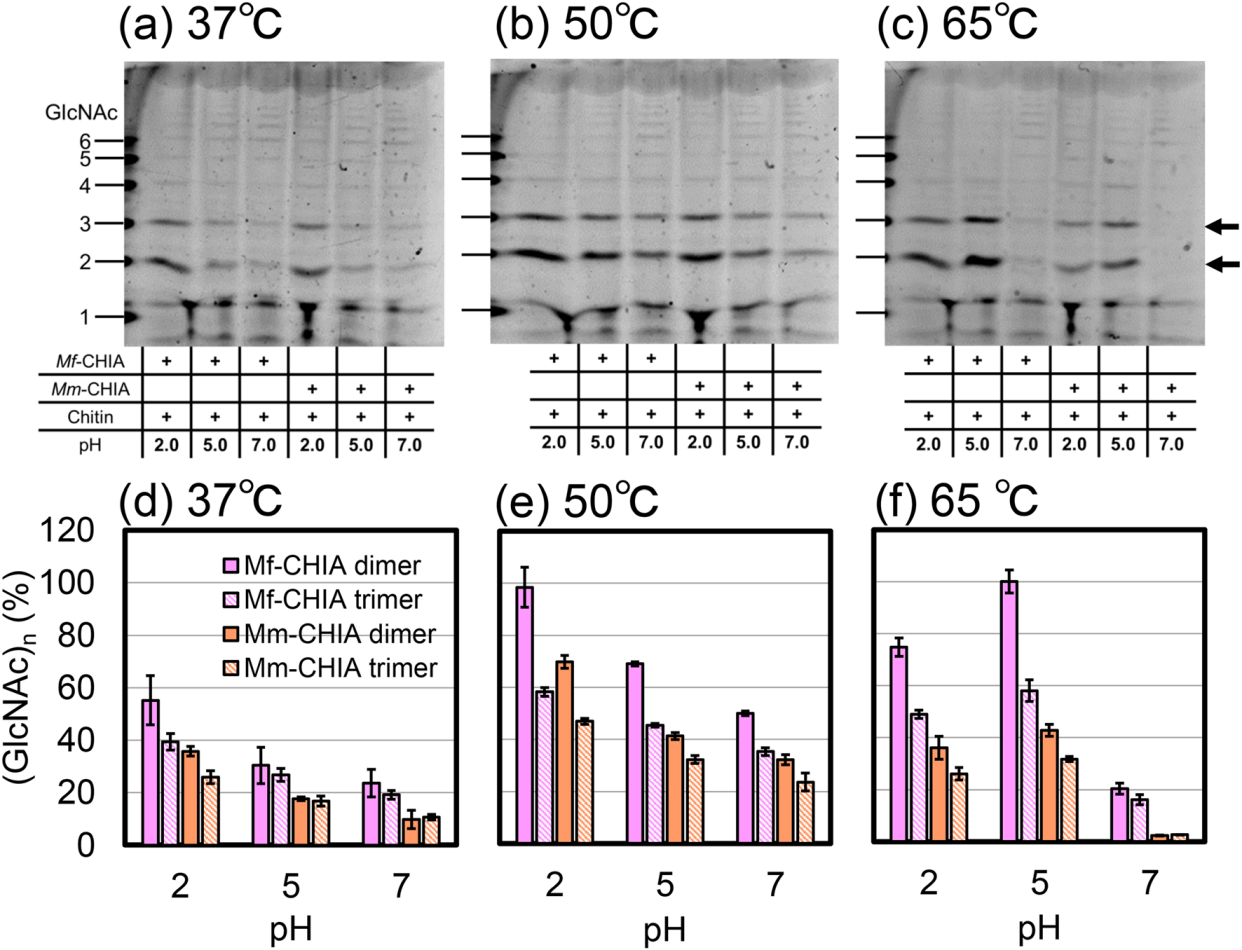
Degradation of polymeric chitin by *Mf*-CHIA and *Mm*-CHIA. Polymeric chitin was incubated with *Mf*-CHIA or *Mm*-CHIA at pH 2.0, 5.0 or 7.0 for 60 min under various temperature conditions. (**a**) 37 °C, (**b**) 50 °C and (**c**) 65 °C. The images of (**a**,**b**,**c**) were cropped from red dotted lines on original full-length gel images shown in Supplementary Fig. S7. The resulting products were analyzed by the FACE method. Chitin oligomers are shown in the left margin as standards. The quantitative data of (GlcNAc)_2_ and (GlcNAc)_3_ are shown (**d**,**e**,**f**). Pink, *Mf*-CHIA; orange, *Mm*-CHIA and filled bars, dimer; hatched bars, trimer. We quantified the (GlcNAc)_2_ and (GlcNAc)_3_ produced by the enzymes and show the relative activity when the *Mf*-CHIA degradation product's peak was set to 100%. Error bars represent mean ± standard deviation from a single experiment conducted in triplicate (Supplementary Fig. S8).

Next, we incubated polymeric chitin (P-CHITN; Megazyme, Bray, Ireland) with *Mf*-CHIA or *Mm*-CHIA using McIlvaine’s buffer at pH 2.0, 5.0 or 7.0 and 37, 50 or 65 ℃. The degradation products were analyzed by fluorophore-assisted carbohydrate electrophoresis (FACE)^38,39^. We quantified the (GlcNAc)_2_ and (GlcNAc)_3_ produced by the enzymes under each pH and temperature condition (Fig. 6 and Supplementary Figs. S8 and S9). We show the relative activity when the *Mf*-CHIA degradation product's peak was set to 100%. At 37 ℃ and 50 ℃, high levels of (GlcNAc)_2_ produced by *Mf*-CHIA were observed at pH 2.0 and pH 5.0 (Fig. 6a,b,d,e and Supplementary Figs. S8a, S8b, S9a and S9b). Most degradation products with *Mf*-CHIA were obtained at pH 2.0, although the optimal pH level was 5.0. On the other hand, at 65 ℃, the optimal pH was 5.0 where both dimer and trimer production peaked (Fig. 6c,f and Supplementary Figs. S8c and S9c). (GlcNAc)_3_ was also produced by both *Mf*-CHIA and *Mm*-CHIA, although it did not reach the amount of (GlcNAc)_2_. Under each condition, *Mf*-CHIA performed more efficiently than *Mm*-CHIA.

## Discussion

Chitinases have been attracting scientific attention due to their association with different pathophysiological conditions^13–25^. In this study, we show robust enzymatic activity as well as pH- and thermostability of *Macaca fascicularis* CHIA (*Mf*-CHIA).

We found that *Mf*-CHIA achieves highest activity at pH 5.0 and remains active at pH 1.0–7.0 (Fig. 2a). We assume that these features can be related to its principal expression and localization in stomach^29^, where the pH shifts from 2.0 to 5.0–7.0 after feeding^40^.

*Mf*-CHIA was three times more active than *Mm*-CHIA under respective optimal pH levels (Fig. 2a) and significantly higher at all tested conditions (Figs. 2 and 3). Expression and activity levels of acidic chitinases are much higher in omnivorous animals in comparison with carnivorous and herbivorous animals^36^. It has been reported that *Mf*-CHIA is 50 times more active than human CHIA^37^. However, pH-dependent profiles of *Mf*-CHIA were similar to those of human CHIA^23,34,37^.

*Mf*-CHIA was most active at 65 ℃ and pH 5.0 (optimal pH) and more efficient in artificial chromogenic (Figs. 2 and 3) and polymeric chitin substrates (Fig. 6) degradation at temperatures (50–70 ℃) above normal body temperature (37 ℃). Moreover, the remarkable stability of this enzyme was demonstrated also in strong acidic environment (Figs. 4a,b, 5a). Thus, we clarified the enzymatic properties of *Mf*-CHIA and identified the inactivating conditions.

Using polymeric chitin, *Mf*-CHIA produced at 37 ℃ and 50 ℃ more degradation products at pH 2.0 than at its optimal pH 5.0 (Fig. 6). The active center (DXXDXDXE motif) in Chia proteins, including *Mf*-CHIA, is thought to have an essential role in substrate binding and catalysis in acidic conditions, with His187 being responsible for the acidic optimum^41^. Interestingly, the optimal condition for polymeric chitin degradation (pH 2.0, 50 ℃) differs from the optimal condition for chromogenic substrate degradation by CHIA (pH 5.0, 65 ℃). The reason for this discrepancy is yet to be revealed and is under further investigation.

In this study, we show a detailed characterization of *Macaca fascicularis* CHIA (*Mf*-CHIA). *Mf*-CHIA seems to be more active toward 4-NP-(GlcNAc)_2_ chromogenic substrate than toward polymeric chitin (Figs. 2, 3 and 6). This is in agreement with a previous report showing that activity variability between experiments using chromogenic substrate and polymeric chitin may be driven by differential substrate specificity^12^.

Chitooligosaccharides have been reported to have various anti-tumor and anti-inflammatory activity while being involved in certain metabolic diseases^42–45^. *Mf*-CHIA activity was high under a broad range of pH and temperature conditions, demonstrating its acid—and thermostability. Our present results also indicate that *Mf*-CHIA has a promising potential to become the enzyme of choice for chitooligosaccharides production for agricultural and medical purposes.

## Methods

### Monkey and mouse total RNAs

The study was designed and carried out in compliance with the ARRIVE guidelines^46^. We purchased crab-eating monkey (*Macaca fascicularis*) total RNA from UNITECH Co., Ltd., Chiba, Japan, and mouse total RNAs (BALB/c mice) from and Takara Bio USA, Inc., Mountain View, CA, USA. We did not use living animals but expressed proteins in *E. coli*. The use of animal-derived total RNAs and all procedures in this study were reviewed and approved by the Recombinant DNA Committee at Kogakuin University.

### *E. coli* expression vectors

We used monkey or mouse stomach total RNAs and reverse transcribed as previously described^29,33^. Coding regions of the mature form of *Macaca fascicularis* CHIA (*Mf*-CHIA) and *Mus musculus* CHIA (*Mm*-CHIA) cDNAs were amplified from the corresponding animal’s cDNAs by PCR using KOD Plus DNA polymerase (Toyobo Co., Ltd, Osaka, Japan) and oligonucleotide primers (Eurofins Genomics, Tokyo, Japan) anchored with the restriction sites for BamHI or XhoI (Supplementary Table S1) as described previously^33^. We obtained monkey and mouse cDNA by reverse transcription of total RNA. Amplified cDNA was digested with BamHI and XhoI and inserted into the pEZZ18 vector. The entire nucleotide sequence of the resulting plasmid DNA (pEZZ18/CHIA/V5-His) was confirmed by sequencing (Eurofins Genomics). Expression of these plasmid DNA in *E. coli* cells led to the production of the mature Protein A-CHIA-V5-His.

### Preparation of the recombinant chitinase proteins expressed in *E. coli*

*E. coli* BL21 (DE3) (Merck Millipore, Tokyo, Japan) was transformed by pEZZ18/pre-Protein A-CHIA-V5-His to express pre-Protein A-*Mf*-CHIA-V5-His or pre-Protein A-*Mm*-CHIA-V5-His proteins. Transformed *E. coli* BL21 (DE3) strains were grown in 3 L LB medium containing 100 µg/mL ampicillin at 37 °C for 18 h. Cells were harvested by centrifugation at 7,000*g* for 20 min at 4 °C. The recombinant protein in the soluble fraction was passed through the IgG Sepharose column (GE Healthcare, Piscataway, NJ, USA) as described previously^33^. The protein-containing fractions were desalted using PD MidiTrap G-25 (GE Healthcare) equilibrated with the TS buffer [20 mM Tris–HCl (pH 7.6), 150 mM NaCl and a protease inhibitor (Complete, Roche, Basel, Switzerland)]. We analyzed the protein fractions using standard SDS-PAGE, followed by Western blot. Separated proteins were transferred to a polyvinylidene fluoride (PVDF) membrane (Immobilon-P, Merck Millipore), which was probed using a polyclonal anti-V5-HRP monoclonal antibody (Invitrogen, Carlsbad, CA, USA). We also conducted SYPRO Ruby staining (Thermo Fisher Scientific, Waltham, MA, USA) according to manufacturer`s instructions. We analyzed and quantified the immunoblots using the Luminescent Image Analyzer (ImageQuant LAS 4000, GE Healthcare). Protein concentration was determined by the Protein Assay (Bio-Rad, Richmond, CA, USA) based on the method of Bradford with bovine serum albumin as the standard.

### Zymography assays

We performed zymography analysis using standard SDS-PAGE gel except for containing 0.1% ethylene glycol chitin (Wako Pure Chemical Industries). Samples were loaded without heat denaturation in SDS-free sample buffer. After electrophoresis, we stained gel using Calcofluor white M2R (Sigma-Aldrich) as described previously^30^. The gels were analyzed using the Luminescent Image Analyzer.

### Chitinase enzymatic assays

We determined chitinolytic activity using 4-nitrophenyl *N*,*N*′-diacetyl-β-D-chitobioside [4-NP-(GlcNAc)_2_, Sigma-Aldrich, St. Louis, MO, USA], at a concentration of 200 µM as described previously^33^. We incubated *Mf*-CHIA or *Mm*-CHIA for 1 h under various conditions (pH 1.0–8.0 and 30–70 °C). The absorbance of the released 4-nitrophenol (4-NP) was measured at 405 nm. A molar extinction coefficient for 4-NP of 17,700 M^−1^ cm^−1^ was used in the calculations. One enzyme unit (U) was defined as 1 μmol of 4-NP liberated from 4-NP-(GlcNAc)_2_ per min at 37 °C at each pH.

### Influence of pH and temperature on the chitinase stability

To determine the pH stability, we incubated *Mf*-CHIA for 1 h at 0 ℃, 37 ℃ or 65 ℃ in 0.1 M Gly-HCl buffer (pH 1.0 to 3.0) and McIlvaine’s buffer (pH 2.0 to 8.0). After the pre-incubation, we measured the residual activity at pH 5.0 in McIlvaine’s buffer, as described above.

For heat stability measurement, we incubated the monkey CHIA in 0.1 M Gly-HCl buffer (pH 2.0, 5.0 or 7.0) for 30 min between 30 and 80 °C. After cooling on ice, we measured the activity in McIlvaine’s buffer (pH 5.0), as described above.

### Degradation of polymeric chitin

All enzymatic reactions using chitin (P-CHITN, Megazyme) (1 mg/reaction) were incubated in a volume of 50 µL containing recombinant *Mf*-CHIA or *Mm*-CHIA at pH 2.0, 5.0 and 7.0. The reaction was initiated by adding the enzyme to the substrate-containing mixture in McIlvaine’s buffer (pH 2.0, 5.0 and 7.0) followed by incubation at 37 °C, 50 °C or 65 °C for 1 h. The degradation products were labeled and separated by fluorophore-assisted carbohydrate electrophoresis (FACE), as described previously^38,39^. We took all gels with the same exposures. *N*-acetyl chitooligosaccharides (Seikagaku Corporation, Tokyo, Japan) were used as a standard.

### Statistical analysis

Biochemical data were compared by Student’s t-test.

## Supplementary Information

Supplementary Information.

## Data Availability

The datasets generated and/or analyzed during the current study are available from the corresponding author on reasonable request.
