# Supplementary material for: Robust chitinolytic activity of crab-eating monkey (Macaca fascicularis) acidic chitinase under a broad pH and temperature range
Source: Sci Rep. 2021 Jul 29;11:15470. doi: 10.1038/s41598-021-95010-w (PMC8322401; doi:10.1038/s41598-021-95010-w)
Supplement: Supplementary file 1 — Supplementary Information. [file 41598_2021_95010_MOESM1_ESM.docx]

Robust chitinolytic activity of crab-eating monkey (*Macaca fascicularis*) acidic chitinase under a broad pH and temperature range

Maiko Uehara^1^, Eri Tabata^1,2^, Mikoto Okuda^1^, Yukari Maruyama^1^, Vaclav Matoska^3^, Peter O. Bauer^3,4^ and Fumitaka Oyama^1, *^

^1^Department of Chemistry and Life Science, Kogakuin University, Hachioji, Tokyo 192-0015, Japan, ^2^Research Fellow of Japan Society for the Promotion of Science (PD), Koujimachi, Chiyoda-ku, Tokyo 102-0083, Japan, ^3^Laboratory of Molecular Diagnostics, Department of Clinical Biochemistry, Hematology and Immunology, Homolka Hospital, Roentgenova 37/2, Prague 150 00, Czech Republic, ^4^Bioinova Ltd., Videnska 1083, Prague 142 20, Czech Republic

Protein A-*Mf*-CHIA-V5-His

617 amino acids, 68,488 dalton

AQHDEAVDNKFNKEQQNAFYEILHLPNLNEEQRNAFIQSLKDDPSQSANLLAEAKKLNDAQAPKVDNKFNKEQQNAFYEILHLPNLNEEQRNAFIQSLKDDPSQSANLLAEAKKLNDAQAPKVDANSSSVPGDPYQLTCYFSNWAQYRPGLGRFMPDDIDPCLCTHLIYAFAGMQNNKITTIEWNDVTLYQAFNGLKNKNSQLKTLLAIGGWNFGTAPFTAMVSTPANRQTFINSVIEFLRQYEFDGLDFDWEYPGSRGSPSQDKHLFTVLVQETREAFEQEAKQSNKPRLLVTAAVAAGISTIQSGYEIPQLSQYLDYIHVMTYDLHGPWEGYTGENSPLYKYPTDTGSNAYLNVDYAINYWKNNGAPAEKLIVGFPAYGHTFLLSNPSDHGIGAPTTGPGPAGPYTRESGFWAYYEICTFLKNGATEVWEANEDVPYAYKGNEWLGYDNTKSFQIKADWLKKNNFGGAMVWAIDLDDFTGTFCNEGKFPLITTLKDALGLQSTSCKAPAQPITPITEAPVTGSVSHSGSSGGSPSDSEFCANKANGLYPDPTDKNAFYNCANGKTFIQHCQAGLVFEASCSCCSWARGHPFEGKPIPNPLLGLDSTRTGHHHHHH

Protein A-*Mm*-CHIA-V5-His

609 amino acids, 67,629 dalton

AQHDEAVDNKFNKEQQNAFYEILHLPNLNEEQRNAFIQSLKDDPSQSANLLAEAKKLNDAQAPKVDNKFNKEQQNAFYEILHLPNLNEEQRNAFIQSLKDDPSQSANLLAEAKKLNDAQAPKVDANSYNLICYFTNWAQYRPGLGSFKPDDINPCLCTHLIYAFAGMQNNEITTIEWNDVTLYKAFNDLKNRNSKLKTLLAIGGWNFGTAPFTTMVSTSQNRQTFITSVIKFLRQYGFDGLDLDWEYPGSRGSPPQDKHLFTVLVKEMREAFEQEAIESNRPRLMVTAAVAGGISNIQAGYEIPELSKYLDFIHVMTYDLHGSWEGYTGENSPLYKYPTETGSNAYLNVDYVMNYWKNNGAPAEKLIVGFPEYGHTFILRNPSDNGIGAPTSGDGPAGPYTRQAGFWAYYEICTFLRSGATEVWDASQEVPYAYKANEWLGYDNIKSFSVKAQWLKQNNFGGAMIWAIDLDDFTGSFCDQGKFPLTSTLNKALGISTEGCTAPDVPSEPVTTPPGSGSGGGSSGGSSGGSGFCADKADGLYPVADDRNAFWQCINGITYQQHCQAGLVFDTSCNCCNWPARGHPFEGKPIPNPLLGLDSTRTGHHHHHH

Supplementary Fig. S1. Recombinant proteins expressed in *E. coli.* Amino acid sequences are color-coded as follows: Yellow, mature form of the truncated Protein A ; pink, crab-eating monkey, *Macaca fascicularis* CHIA (*Mf*-CHIA); orange, mouse, *Mus musculus* CHIA (*Mm*-CHIA); green, V5 - His sequence.


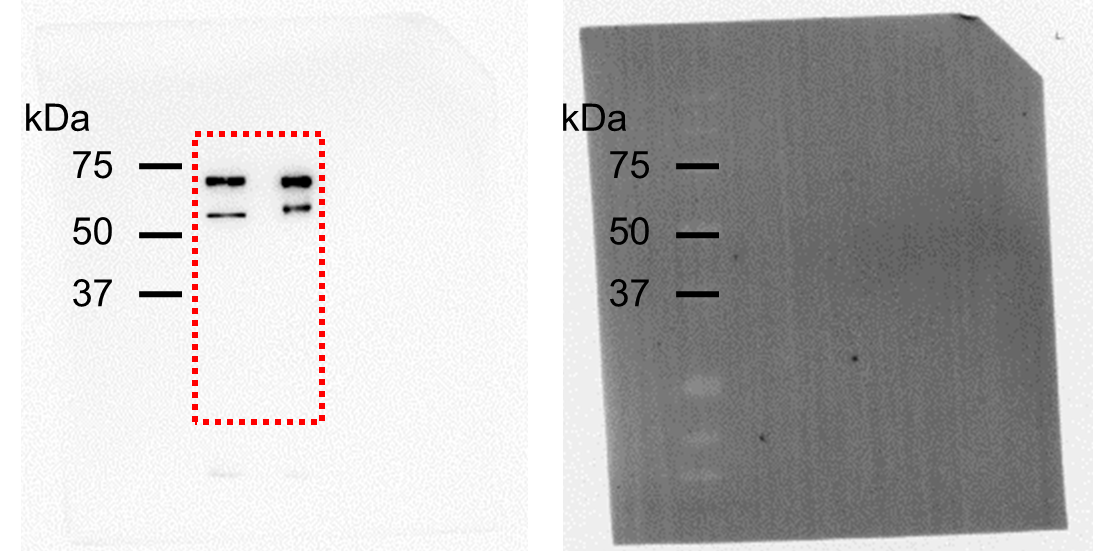


Supplementary Fig. S2. Full-length gel image for Fig. 1b. Full-length gel image of SDS-PAGE and Western blot using anti-V5-HRP monoclonal antibody (left) and its molecular weight markers (right).


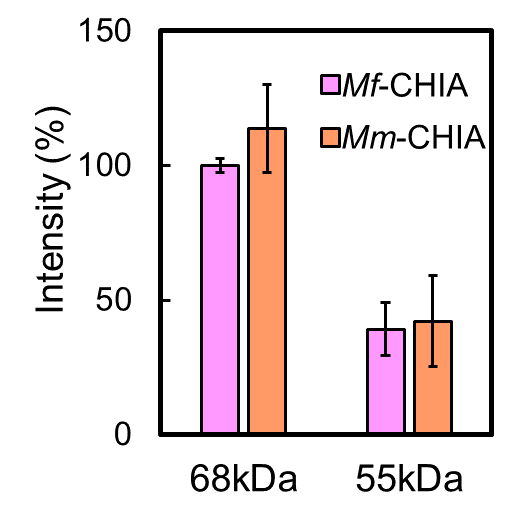

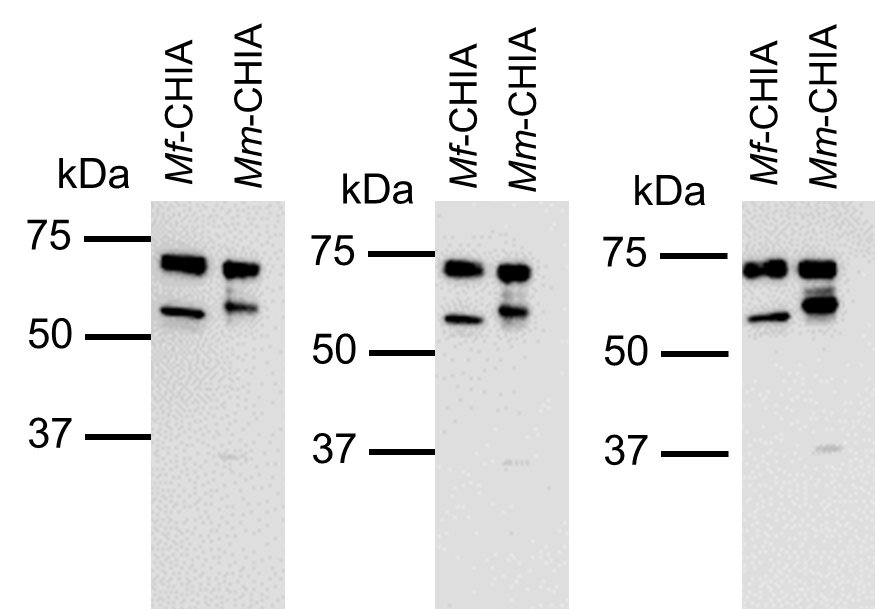


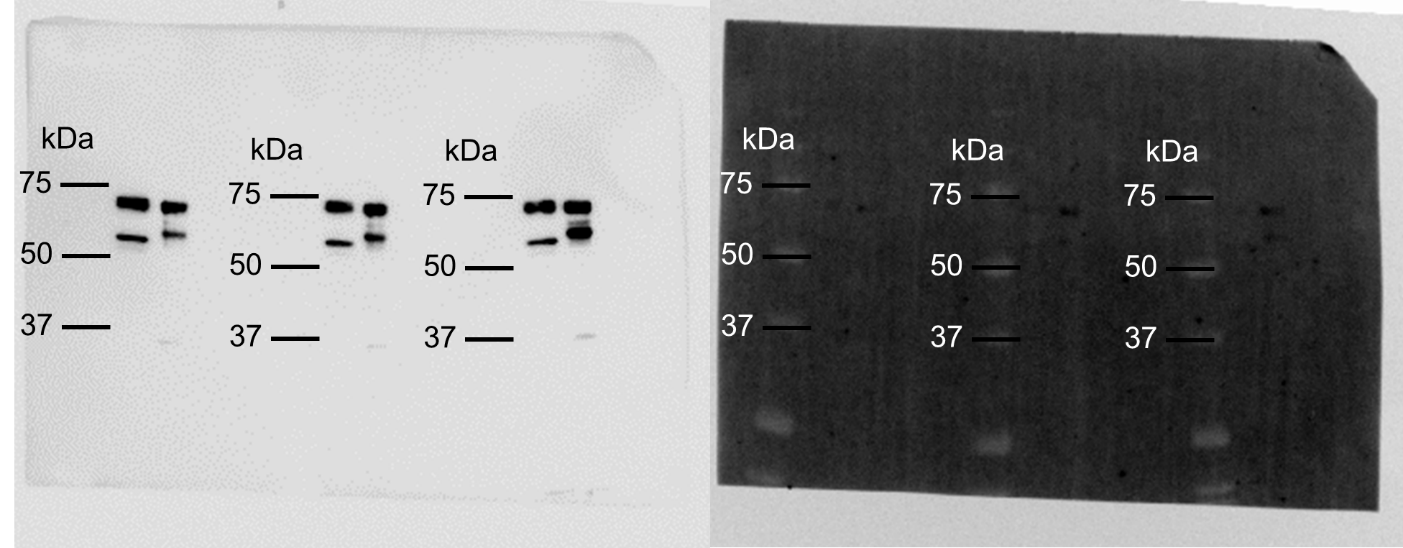


Supplementary Fig. S3. Quantification of the proteins (related to Fig. 1b). Recombinant proteins were separated by SDS PAGE and analyzed by Western blot as described in Fig. 1b. Quantification shows relative values of signal intensities. The signal intensity of monkey CHIA was set to 100%. Error bars represent mean ± standard deviation from a single experiment conducted in triplicate. Lower panels were full-length images of SDS-PAGE and Western blot using anti-V5-HRP monoclonal antibody (left) and its molecular weight markers (right).


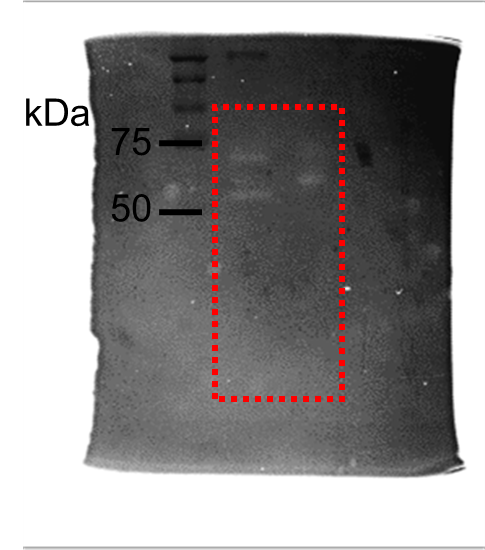


Supplementary Fig. S4. Full-length gel image for Fig. 1c. Full-length gel image of SDS-PAGE and Zymography.


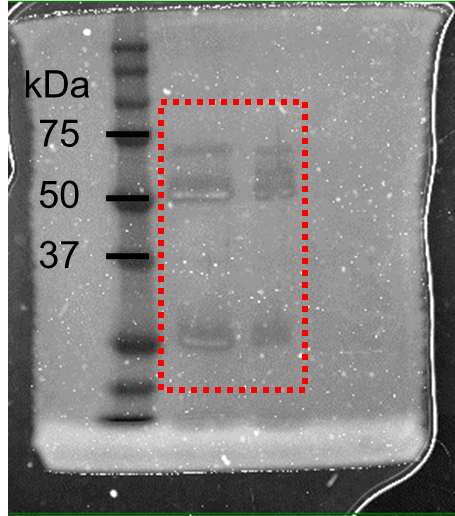


Supplementary Fig. S5. Full-length gel image for Fig. 1d. Full-length gel images of SDS-PAGE and SYPRO ruby staining.

Supplementary Fig. S6. Relation between the initial substrate and consumed substrate. X-axis, concentration of the initial substrate; y-axis, concentration of consumed substrate. The enzymes concentration was set to provide a 20% rate of substrate consumption as compared to the initial solution. We measured the enzymatic activity of *Mf*-CHIA at 37℃ and pH 5.0 for 1 hour. When we set the initial concentration to 200 μM, the percent of the consumed substrate was around 15%.


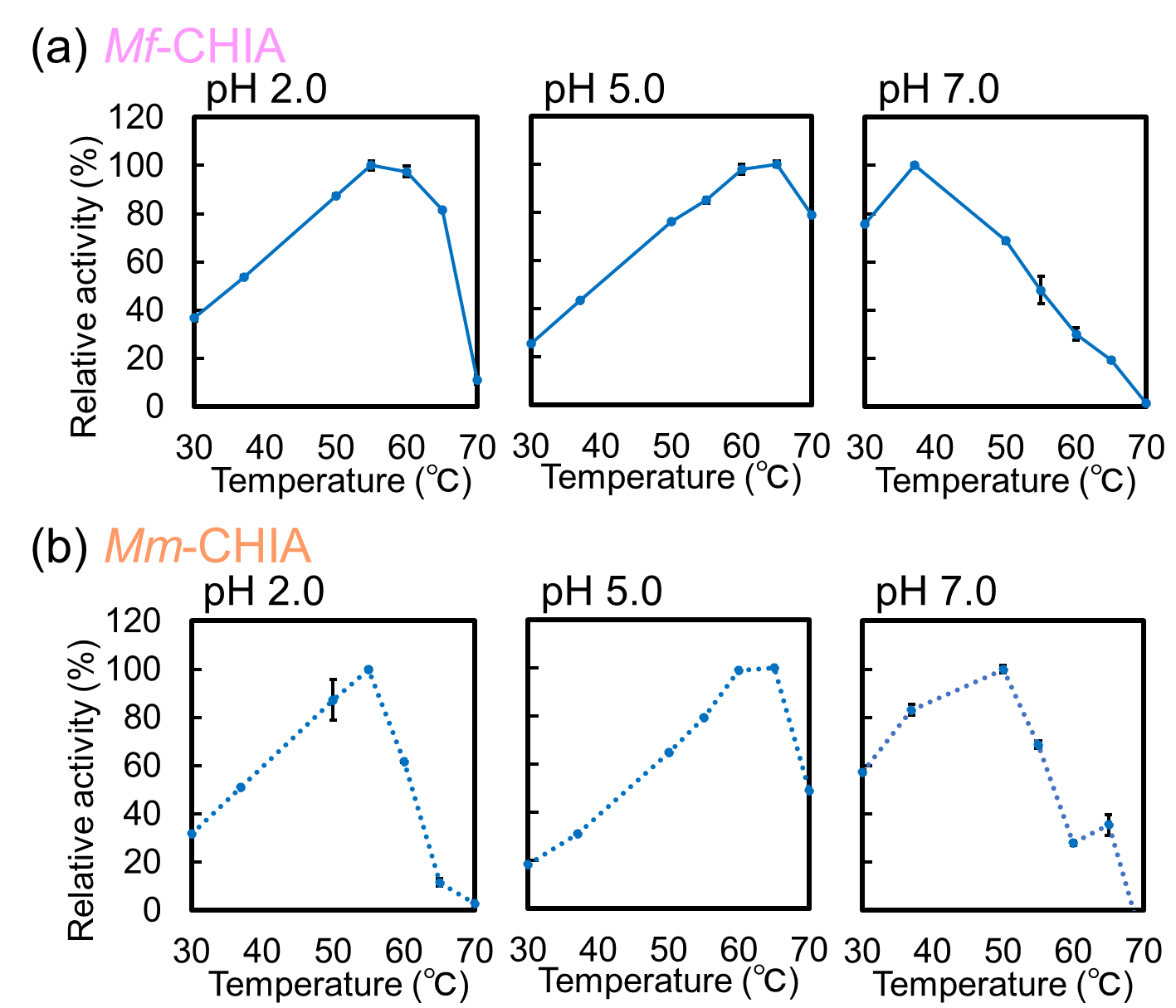


Supplementary Fig. S7. Optimal temperatures of *Mf*-CHIA and *Mm*-CHIA. (a) *Mf*-CHIA, solid lines; (b) *Mm*-CHIA, dotted lines. We showed the relative activities when the highest activities of *Mf*-CHIA or *Mm-CHIA* under each pH conditions, respectively, were set to 100%.


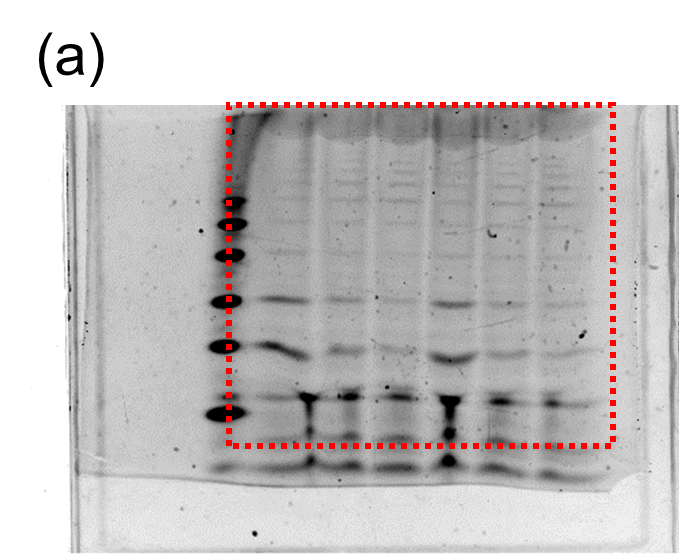


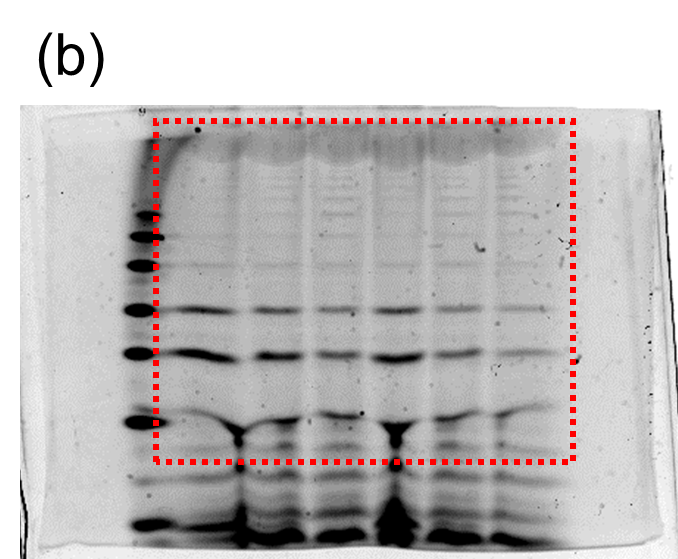


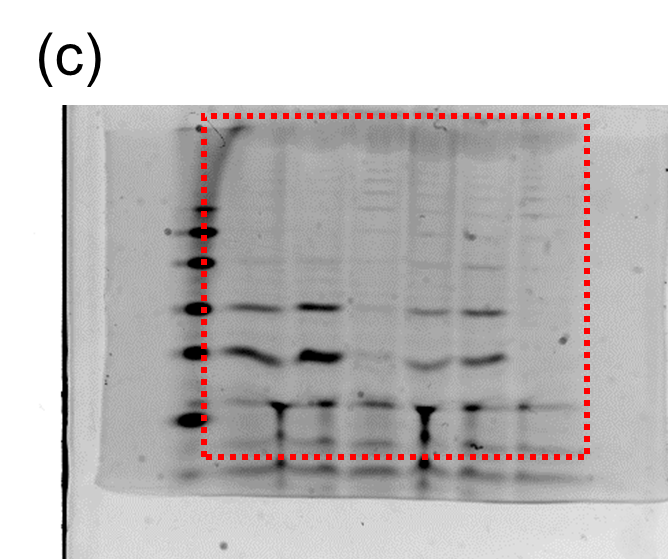


Supplementary Fig. S8. Full-length gels for Fig. 6. Full-length gel images of the FACE method in Fig. 6a, 6b and 6c. (a) Fig. 6a, (b) Fig. 6b and (c) Fig. 6c.


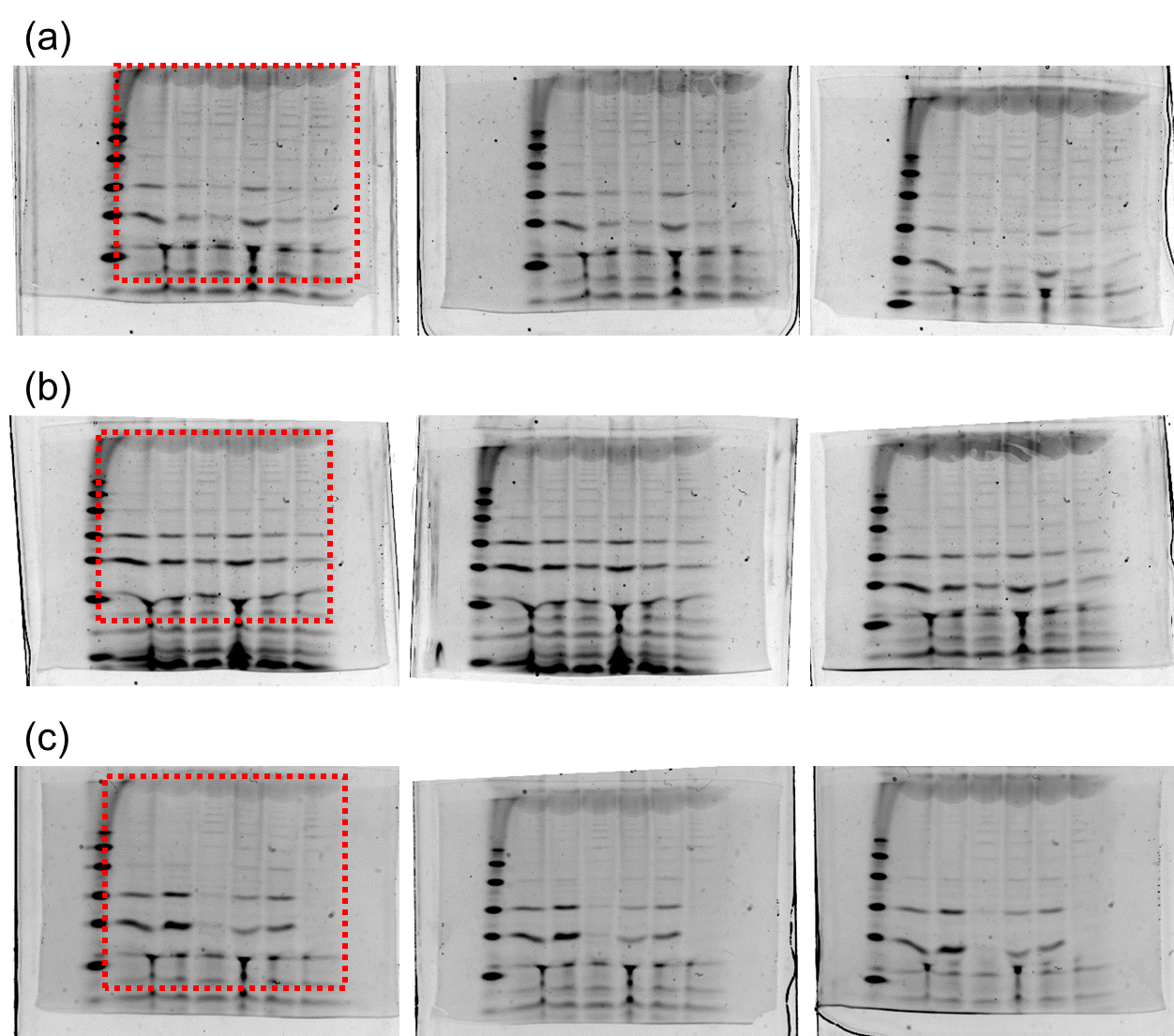


Supplementary Fig. S9. Gels for quantitative analysis shown in Fig. 6d, 6e and 6f. Gel images of FACE method for Fig. 6. (a) 37℃, (b) 50℃ and (c) 65℃. Experiments were conducted in triplicate. Signal intensities of (GlcNAc)_2_ and (GlcNAc)_3_ in these gel images are quantified and shown in Fig. 6d, 6e and 6f.


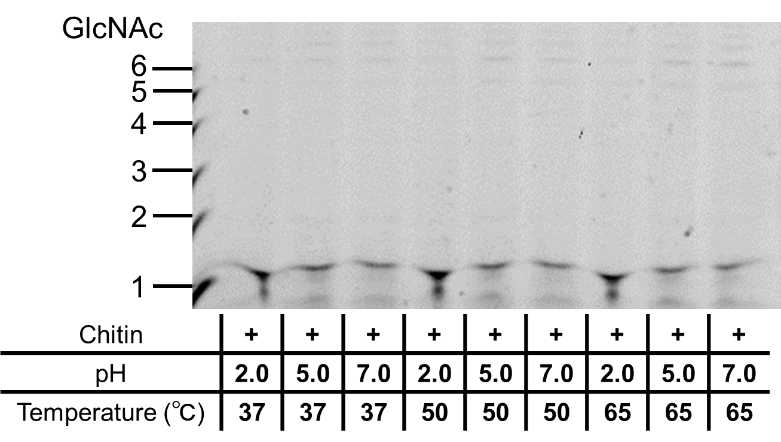
　
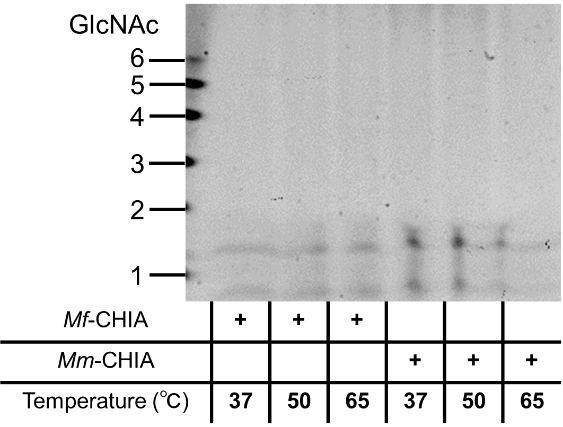


Supplementary Fig. S10. Gels loaded with substrate, *Mf*-CHIA or *Mm*-CHIA. We show gel images with exception of enzyme (left) and exception of substrate (right).

Supplementary Table S1. List of PCR primers for preparation of recombinant proteins.

| Primer | Sequence |
| --- | --- |
| *Mf*-CHIA BamHI Fw | CGCGGAACCCGGATCCGTACCAGCTGACATGCTACTTCTCCA |
| *Mf*-CHIA XhoI Rv | GTGACCTCGAGCCCAGCTGCAGCAGGAGCAGGAGGCT |
| *Mm*-Chia EcoRI Fw | CATGGAATTCGTACAATCTGATATGCTATTTCACC |
| *Mm*-Chia SalI_BGH Rv | AGGGGTCGACTAGAAGGCACAGTCGAGGCTGATCA |
